# Microbial Corrosion Resistance and Antibacterial Property of Electrodeposited Zn–Ni–Chitosan Coatings

**DOI:** 10.3390/molecules24101974

**Published:** 2019-05-22

**Authors:** Xiaofan Zhai, Yadong Ren, Nan Wang, Fang Guan, Maria Agievich, Jizhou Duan, Baorong Hou

**Affiliations:** 1 CAS Key Laboratory of Marine Environmental Corrosion and Bio-Fouling, Institute of Oceanology, Chinese Academy of Sciences, No.7 Nanhai Road, Qingdao 266071, China; ryd19941106@163.com (Y.R.); wnan123@126.com (N.W.); guanfang@qdio.ac.cn (F.G.); houbr@qdio.ac.cn (B.H.); 2 School of Chemical Engineering, Qingdao University of Science and Technology, 53 Zhengzhou Road, Qingdao 266042, China; 3 Open Studio for Marine Corrosion and Protection, Pilot National Laboratory for Marine Science and Technology (Qingdao), No. 1 Wenhai Road, Qingdao 266235, China; 4 Center for Ocean Mega-Science, Chinese Academy of Sciences, 7 Nanhai Road, Qingdao 266071, China; 5 Institute of Living Systems, Immanuel Kant Baltic Federal University, 14 A. Nevskogo ul., 236016 Kaliningrad, Russia; myamina@mail.ru

**Keywords:** Zn–Ni alloy, chitosan, microbial corrosion resistance, antibacterial

## Abstract

Microbial corrosion is a universal phenomenon in salt water media such as seawater and wastewater environments. As a kind of efficient protective metal coating for steel, the damage of the Zn–Ni alloy coating was found to be accelerated under microbial corrosive conditions. To solve this problem, chitosan, which is considered a natural product with high antibacterial efficiency, was added to Zn–Ni electrolytes as a functional ingredient of electrodeposited Zn–Ni–chitosan coatings. It was found that the addition of chitosan significantly and negatively shifted the electrodeposition potentials and influenced the Ni contents, the phase composition, and the surface morphologies. By exposing the coatings in a sulfate-reducing bacteria medium, the microbial corrosion resistance was investigated. The results showed that compared to the Zn–Ni alloy coating, Zn–Ni–chitosan coatings showed obvious inhibiting effects on sulfate-reducing bacteria (SRB) and the corrosion rates of these coatings were mitigated to some degree. Further research on the coatings immersed in an *Escherichia coli*-suspended phosphate buffer saline medium showed that the bacteria attachment on the coating surface was effectively reduced, which indicated enhanced antibacterial properties. As a result, the Zn–Ni–chitosan coatings showed remarkably enhanced anticorrosive and antibacterial properties.

## 1. Introduction

Zn-based coating electrodeposited on steel is considered one of the most effective and economical methods for providing efficient and reliable corrosion protection [1]. Among all the Zn-based coatings, researchers found that by alloying elements of group VIII (i.e., Fe, Co, Ni), Zn-based coatings could obtain significantly enhanced properties [2,3]. Additionally, in an aquatic environment, the Zn–Ni coatings possess relatively high corrosion resistance and good mechanical properties, which offers an important eco-friendly alternative to toxic Cd coatings [4].

However, the protection performances of the Zn–Ni coatings were still found to be unsatisfactory when applied in a marine environment, which is highly biotic. The most serious problems are considered to be microbiological-induced corrosion (MIC) and biofouling [5,6,7]. In a natural aquatic environment, microorganisms are spontaneously attached to material surfaces, resulting in microbial corrosion and biofouling [8,9,10]. As a kind of anaerobe, sulfate-reducing bacteria (SRB) is considered one of the most significant bacteria responsible for MIC. SRB reduces SO_4_^2−^ to S^2−^ [11,12]. Furthermore, in the biofouling process, the attachment of bacteria, which leads to biofilm formation, is reported to be the most significant stage [8]. As a result, making the Zn-Ni coating a biocide that is inhibitive to bacteria is considered to be the main task for MIC and biofouling problems in marine environments.

During the last decades, in order to solve the problems caused by microorganisms, many kinds of biocides such as organic compounds and metal irons in metal substrates have been applied and they have shown obvious effects [13,14]. However, most of these biocides were based on toxic principles, which led to serious environmental problems. Following environmentally friendly principles, green biocide components such as natural polysaccharide chitosan can be applied. Based on the previous literature [15,16], it has been determined that the cationic amino groups in the chitosan molecule are able to interrupt the bacterial membrane and then disrupt mass transport across bacterial cells, which leads to bacterial death. Due to its green biocide characteristic [17], chitosan has been made into a composite material for many kinds of coatings [18,19,20]. As reported, chitosan can be deposited to form coatings with antibacterial properties using chemical deposition and electrodeposition [21,22]. Furthermore, it is found that Ag or ZnO nanoparticles, phosphate, and hydroxyapatite can be made into a composite material with chitosan to form coatings with enhanced antibacterial properties [23,24,25].

To improve the corrosion resistance of Zn–Ni coatings suffering from MIC and biofouling in marine environments, this study attempts to add chitosan to Zn–Ni coatings to make the resultant coating microbial-corrosion resistant and antibacterial and to provide more effective protection for steel constructions. Because SRB is the most corrosive type of bacteria, and *Escherichia coli* (*E. coli*) is a typical bacteria used to detect colonization [26,27], these two kinds of bacteria were employed as an evaluation medium in this research. The main points of the present investigation are studies of the electrodeposition of Zn–Ni–chitosan coatings, the influence of chitosan on the structure of the coatings, and an evaluation of the corrosion resistance and antibacterial properties in the SRB and *E. coli* mediums.

## 2. Results and Discussion

### 2.1. Influence of the Chitosan on the Coating Electrodeposition

**Figure 1:**
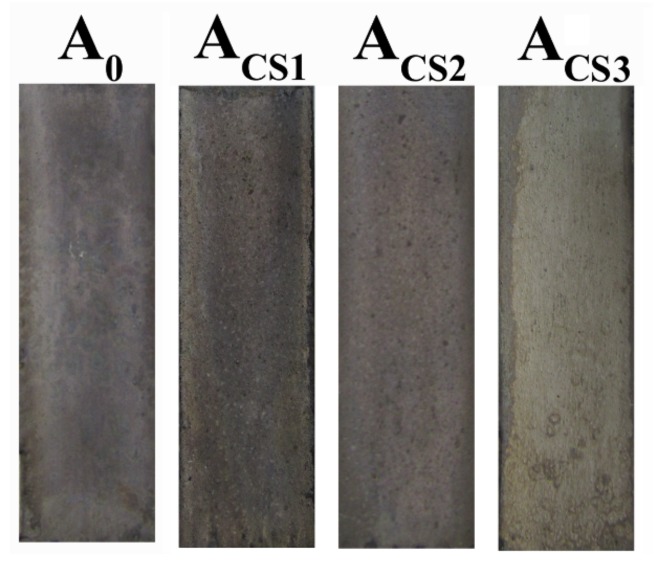
Optical images of the electrodeposited coatings.

Coatings electrodeposited from sulfate electrolytes with 0 g·L^−1^, 0.2 g·L^−1^, 0.6 g·L^−1^, and 1.0 g·L^−1^ of chitosan were named A_0_, A_CS1_, A_CS2_, and A_CS3_, respectively. The optical images of the electrodeposited A_0_, A_CS1_, A_CS2_, and A_CS3_ are shown in Figure 1. The pure Zn-Ni coating A_0_ revealed a smooth but dark surface, while A_CS1_ and A_CS2_ showed similar dark but rough surfaces. When the added chitosan concentration went up to 1.0 g·L^−1^, the surface of the resultant coating was bright and smooth, revealing good apparent properties.

**Figure 2:**
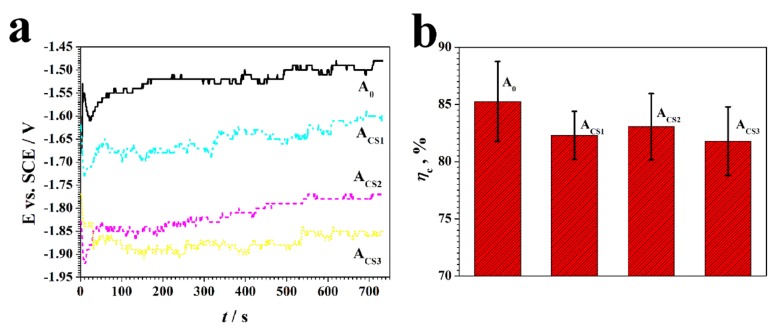
(**a**) The electrodepositing potential variation and (**b**) the current efficiency of the coatings.

To further investigate the influence of chitosan on the electrodeposition process, the depositing potential was monitored. The potential variation is shown in Figure 2a. During the deposition, the potential of all the coatings was unstable with small fluctuations, which could be attributed to the side reaction of hydrogen evolution. The resultant hydrogen bubbles covering the surfaces of the coatings resulted in a decreased effective electrodepositing area, so the production and escape of hydrogen gas caused the potential fluctuations [28]. The depositing potential of A_0_ remained at about −1.55 V vs. SCE, while the Zn–Ni-chitosan coatings showed more negative depositing potentials than those of A_0_. As the adding concentration increased, the depositing potentials of A_CS1_, A_CS2_, and A_CS3_ negatively shifted to 15 mV, 25 mV, and 35 mV, respectively. The negative shifts caused by chitosan could be attributed to the adsorption. The chitosan molecule adsorbed onto the electrodepositing surfaces and hindered the electrodeposition [29]. Therefore, the potential turned negative under a stable current, which also accounted for the more obvious negative shifts in the electrolytes with higher concentrations of chitosan.

**Figure 3:**
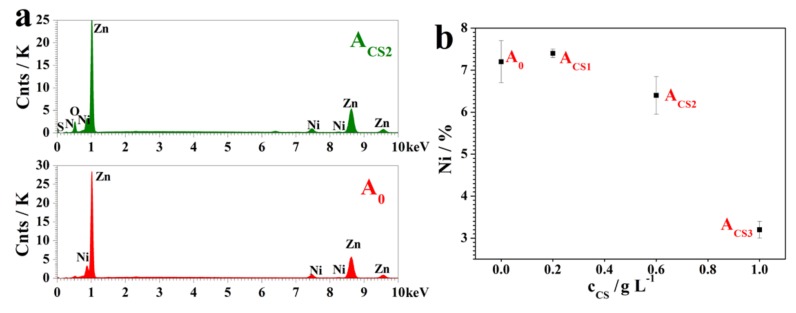
(**a**) The energy dispersive spectroscopy (EDS) results of A_0_ and A_CS2_, and (**b**) The Ni contents of the electrodeposited coatings.

To evaluate the power transferring effectiveness, the cathodic current efficiency, *η*_c_, was calculated based on the Ni content obtained by energy dispersive spectroscopy (EDS, shown in Section 2.2, Figure 3). The variation of *η*_c_ is shown in Figure 2b. The addition of chitosan decreased the *η*_c_ value slightly, which could be mainly attributed to the negative shifts of the depositing potential. Under relatively high negative potential, the evolution of hydrogen was strongly accelerated and the electrodeposited surface was covered, which inhibited the Zn^2+^ and Ni^2+^ reduction [29]. However, the *η*_c_ values of all coatings showed no significant differences (*p* > 0.05) and remained relatively high at over 80%, illustrating the high electric conversion efficiencies, which was an important practical indicator.

### 2.2. The Influence of Chitosan on the Coating Structure

Because the Ni content in the Zn–Ni alloy coatings was considered one of the most important parameters indicating the coating properties, EDS was performed to analyze the elemental composition of these electrodeposited coatings. The EDS results for A_0_ and A_CS2_ are shown in Figure 3a, where A_CS1_ and A_CS3_ showed similar results to A_CS2_. As shown in Figure 3b, by measuring the content of Ni atoms in these Zn–Ni coatings through EDS, it was found that the chitosan obviously changed the Ni content in the coatings. The Ni content of A_0_ was 7.2 at.%, while the Ni content of A_CS1_ was 7.4 at.%, which was almost the same as that of A_0_. As the concentration increased, the Ni contents of A_CS2_ and A_CS3_ decreased to 6.4 at.% and 3.2 at.%, respectively. As a result, chitosan added at low concentrations of 0.2 g·L^−1^ would slightly increase the Ni content, which could lead to enhanced corrosion resistance. However, chitosan added at high concentrations of 0.6 g·L^−1^ and 1.0 g·L^−1^ decreased the Ni contents and could lead to low corrosion resistance. The influence of chitosan on the Ni content may have contributed to the chelation effect of chitosan molecules with Zn^2+^ during electrodeposition. As the addition concentration increased, chitosan absorbed on the electrodepositing surface, attracting more Zn^2+^ instead of Ni^2+^ participating in the electrodeposition.

**Figure 4:**
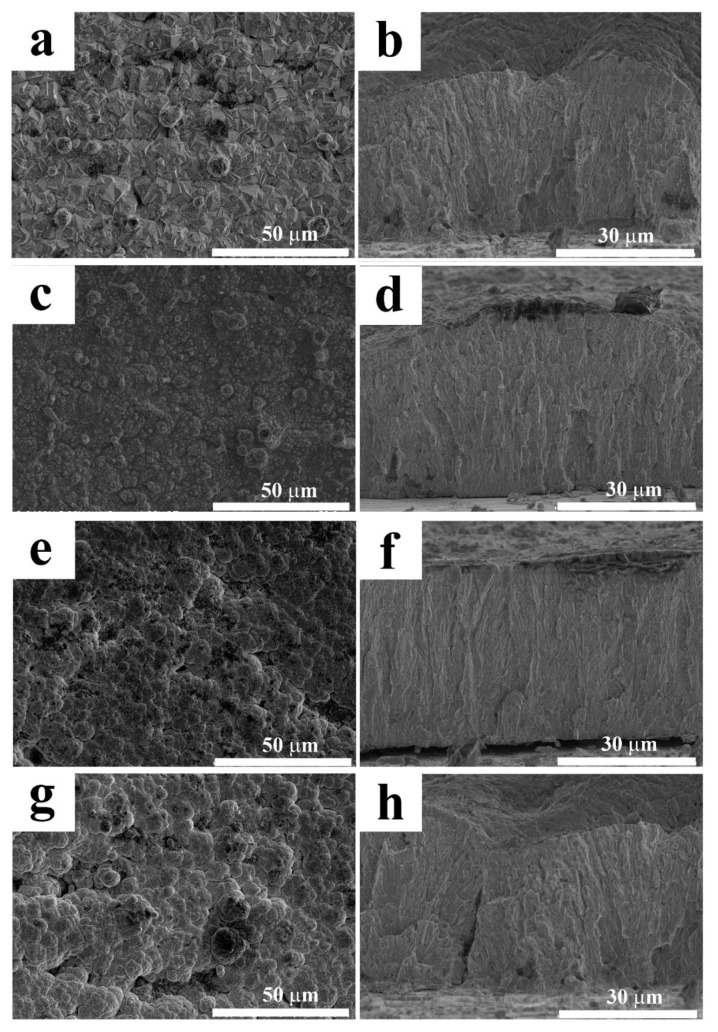
SEM images of the surfaces and cross-sections of (**a**,**b**) A_0_, (**c**,**d**) A_CS1_, (**e**,**f**) A_CS2_, and (**g**,**h**) A_CS3_.

The morphologies of these electrodeposited coatings were observed with a scanning electron microscopy (SEM), as shown in Figure 4. The addition of chitosan to the electrolytes had an enormous influence on the surface morphologies of the coatings. The A_0_ coating showed hexagonal crystals as well as bits of spherical crystals [30]. These spherical crystals also proved to contain higher Ni content than the basal hexagonal crystals [31]. The A_CS1_ coating showed a compact surface with similar spherical crystals to A_0_ and A_CS2_, and A_CS3_ showed obviously different morphologies with bubble-like surfaces. Additionally, A_0_, A_CS2_, and A_CS3_ showed surface defects, while only A_CS1_ revealed a compact surface. The disappearance of the spherical crystals may have contributed to the decrease of Ni content in both coatings. The images of the cross sections of the coatings are provided in Figure 4. It was found that the thicknesses of the coatings were measured as 25 μm–30 μm because of their rough surfaces.

**Figure 5:**
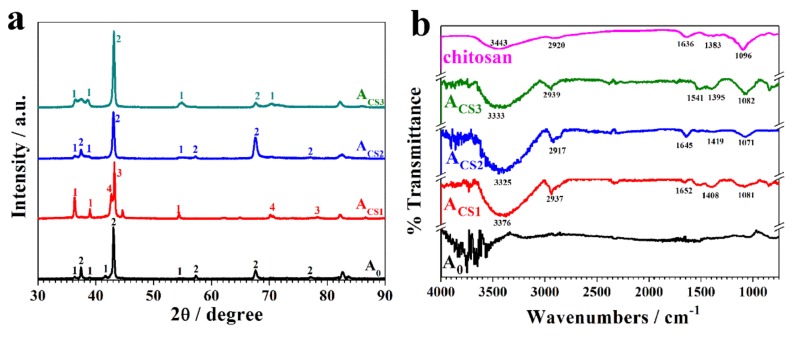
(**a**) XRD patterns and (**b**) the FT-IR spectrum of A_0_, A_CS1_, A_CS2_, A_CS3_, and chitosan. 1: Reflection peaks of the Zn crystal; 2: Reflection peaks of the η-phase Zn–Ni alloy crystal; 3: Reflection peaks of the γ-phase Zn-Ni alloy crystal; 4: Reflection peaks of the δ-phase Zn-Ni alloy crystal.

The crystal structure of the Zn-Ni alloy coatings was strongly influenced by the Ni contents, so the crystal structures were analyzed with X-ray diffraction (XRD, Figure 5a). The main phase of A_0_ was found to be the Zn hexagonal platelets and the hexagonal η phase Zn–Ni alloys with relatively low Ni content, which corresponded to the morphology of A_0_. Because A_CS1_ contained a higher content of Ni than A_0_, the crystal structure changed significantly. The orientations of the pure zinc were enhanced, the orientations of the η phase disappeared, and the orientations of the δ (Ni_3_Zn_22_) and γ (Ni_5_Zn_21_) phases showed up. These alterations could be attributed to the increase in the Ni content. As the added chitosan concentration increased, the alterations of the pure zinc increased and orientations of the η phase decreased, which was also caused by the decrease in the Ni content in the coatings.

To investigate the existence of chitosan in the coatings, Fourier transform infrared spectroscopy (FT-IR) was performed and analyzed based on the EDS results. Figure 3a shows that only Zn and Ni elements were found on the A_0_ coating, while the C, O, and N elements that came from chitosan showed up on the A_CS2_ coating. Based on these elemental analyses, the FT-IR spectrum of A_CS2_ illustrated similar absorption peaks to the chitosan in Figure 5b. All the representative peaks of chitosan were as follows: (1) The peak at approximately 3443 cm^−1^ represented the mixed absorption of the hydrogen bond of –OH and –NH_2_. (2) The peak at 2920 cm^−1^ resulted from the stretching vibrations of the C–H bond in the carbon chain. (3) The peak at 1636 cm^−1^ corresponded to the bending vibration from the –NH_2_ group. (4) The peak appearing at 1383 cm^−1^ derived from the bending vibration of the C–H bond and the deformation vibration of –CH_3_. (5) The peak at 1096 cm^−1^ represented the mixed absorption of the in-plane deformation vibration of the O–H bond and the stretching vibrations of the C–O bond. All these peaks were found on the coatings A_CS1_, A_CS2_, and A_CS3_, indicating that the entire and effective chitosan molecule was found in the coating. Additionally, wave shifts of the representative peaks were observed, indicating that chitosan could exist in the coatings through a chelating effect.

### 2.3. Corrosion Resistance of the Coatings

**Figure 6:**
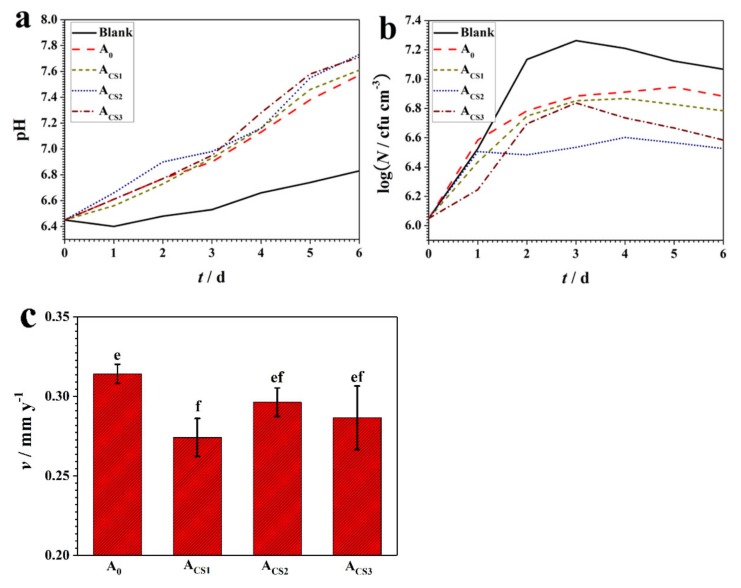
(**a**) The pH value and (**b**) the bacterial concentration variations of the SRB medium with the coatings exposed for six days; (**c**) the corrosion rates of the coatings after six days of exposure in the SRB medium, means with letters e and f denoting significant differences (*p* < 0.05).

To investigate the corrosion resistance of the coatings, they were exposed to an SRB medium for six days. When growing and metabolizing, the SRB produced organic metabolic products, short-chain fatty acids, and the inorganic metabolic product H_2_S, which is acidic. Therefore, to monitor the SRB growth in the surrounding environment, the pH and the bacterial concentration were measured. These measurements are shown in Figure 6a,b.

As illustrated in Figure 6a, the pH of a blank SRB medium without exposed coatings remained faintly acidic, at a pH of approximately 6.5–6.7 during six days of exposure. However, the pH value of the SRB medium with coatings turned alkalescent due to the dissolved components that inhibited the SRB metabolism and growth. The pH value of the SRB medium with A_0_ increased slightly over six days of exposure and reached 7.4, while the pH value of the SRB medium with coatings increased. The pH values of the A_CS2_ and A_CS3_ exposed to the SRB medium shifted most obviously to approximately 7.7–7.8 with an increment of 1.3 over six days, illustrating the most effective inhibition on the SRB metabolism and growth.

The bacterial concentration of the SRB mediums with various coatings over six days of exposure is shown in Figure 6b. The blank SRB medium revealed a standard growth curve and the bacterial concentration reached 10^7.2^ cfu cm^−3^ for a stable period. The SRB medium with A_0_ showed a stable period at the bacterial concentration of 10^6.9^ cfu cm^−3^, which was slightly lower than that of the blank medium due to the dissolved Zn^2+^. The SRB mediums with Zn–Ni–chitosan coatings showed an obvious decrease in bacterial concentration. In particular, for the mediums with A_CS2_ and A_CS3_ over six days of exposure, the bacterial concentration reached 10^6.5^–10^6.6^ cfu cm^−3^. Compared to the blank medium, the sterilizing rates of A_CS2_ and A_CS3_ reached over 80%. Meanwhile, the bacterial concentration of the mediums with A_CS1_ remained relatively low at 10^6.7^ cm^−3^ over six days of exposure, revealing a sterilizing rate of about 70%.

The variations in the pH and the bacterial concentration revealed that the Zn–Ni–chitosan coatings showed a more effective inhibition effect on SRB than the Zn–Ni alloy coating, while the A_CS2_ and A_CS3_ showed the best inhibition effect.

The corrosion rates of the coatings were measured over six days of exposure in the SRB medium, and these rates are shown in Figure 6c. The corrosion rate of A_0_ was calculated to be 0.32 mm y^−1^. The corrosion rates of the chitosan-added alloy coatings decreased by varying degrees. The A_CS2_ and A_CS3_ showed relatively low corrosion rates of 0.30 mm y^−1^ and 0.29 mm y^−1^, respectively. The corrosion rate of A_CS1_ was measured to be 0.27 mm y^−1^, representing the highest corrosion resistance. The enhanced corrosion resistance of the Zn–Ni–chitosan coatings could be attributed to the inhibition of SRB by the chitosan-added coatings.

**Figure 7:**
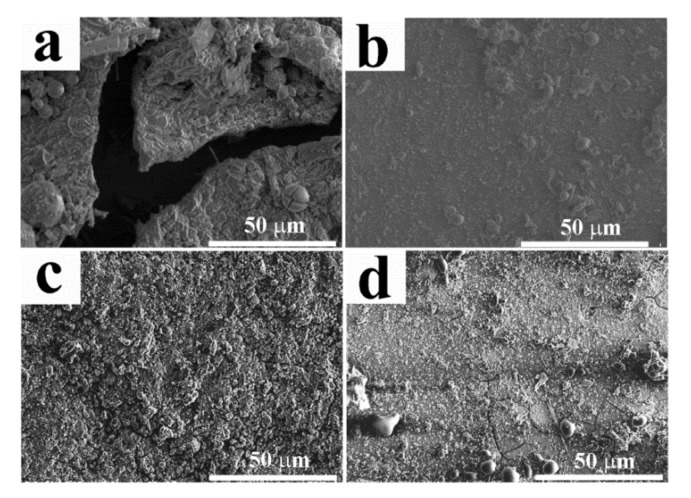
SEM images of (**a**) A_0_, (**b**) A_CS1_, (**c**) A_CS2_, and (**d**) A_CS3_ after corrosion.

To further evaluate the corrosion behavior of the coatings, the SEM images of the coatings with the corrosion products dislodged are shown in Figure 7. After six days of exposure, A_0_ began to break and crack, revealing a rather poor anticorrosive property. Rough surfaces caused by corrosion appeared on A_CS2_ and A_CS3_. For A_CS3_, subtle cracks were found. In contrast, A_CS1_ showed a rather intact and smooth surface, revealing the best anticorrosive property, which corresponded to the corrosion rate results. The relatively high corrosion resistance of A_CS1_ could be attributed to its higher Ni content compared to other chitosan added coatings.

### 2.4. Antibacterial Properties of the Coatings

**Figure 8:**
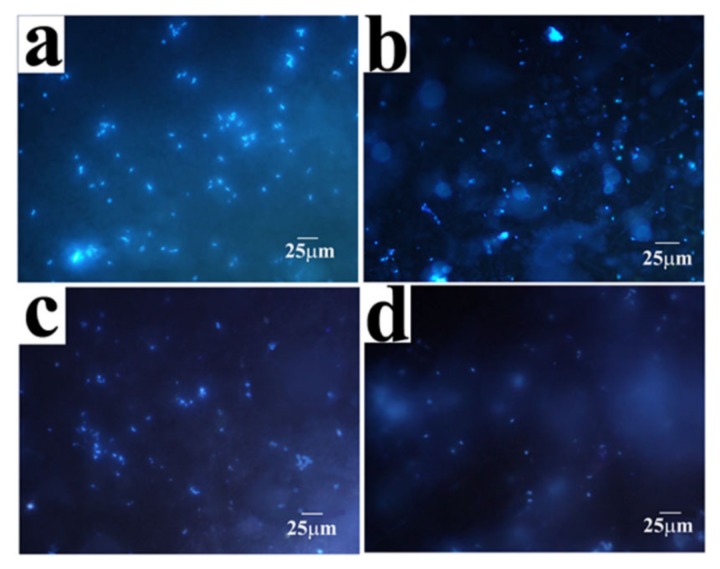
Fluorescence microscopy images of (**a**) A_0_, (**b**) A_CS1_, (**c**) A_CS2_, and (**d**) A_CS3_ after 24 h of exposure in PBS medium suspended with 10^6^ cfu mL^−1^ *E. coli.*

The antibacterial property of the coatings was evaluated in a PBS medium containing 10^6^ cfu mL^−1^ of *E. coli*. After 24 h of immersion, the bacteria attachment was observed with fluorescence staining. The blue points in the fluorescence images represent the bacteria colonized on the coating surface. As shown in Figure 8, many bacteria bodies were attached to the A_0_ surfaces. Multiple bacterial colonies could also be found, which were considered to be the previous step for an intact biofilm. However, only a few bacterial bodies were colonized on the Zn–Ni–chitosan coatings, indicating an original stage of attachment, which was far from a biofilm. As the increased concentration of chitosan to the electrolytes increased, the coverage of the attached bacteria on the coatings decreased.

**Figure 9:**
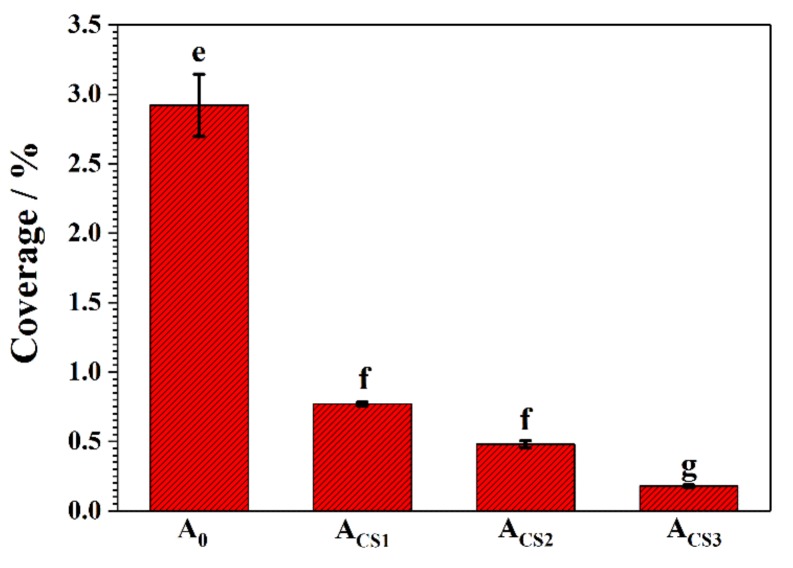
Coverage of bacteria attached to A_0_, A_CS1_, A_CS2,_ and A_CS3_ after 24 h of exposure in a PBS medium suspended with 10^6^ cfu mL^−1^ *E. coli.* Means with letters e, f and g denoting significant differences (*p* < 0.05)

After 24 h of immersion, the coverage of attached bacteria on the coatings was analyzed, as shown in Figure 9. Generally, the attached bacterial coverage of the coatings decreased dramatically as the electrolyte concentration increased. The coverage of the attached bacteria on the A_0_ surface reached 2.92%, which was relatively high when compared to the chitosan added coatings. The coverage of the attached bacteria on A_CS1_, A_CS2,_ and A_CS3_ was 0.77%, 0.48%, and 0.18%, respectively. For A_CS3_, the attached bacteria decreased to 93% compared to A_0_, revealing the best antibacterial property. These results revealed that the existence of chitosan in the coatings could effectively inhibit the adhesion of bacteria to the coating surface. This phenomenon indicated that when exposed to a microbial medium, Zn–Ni–chitosan coatings could first inhibit the bacterial attention, then release chitosan into the medium to inhibit the bacterial growth and metabolism, and finally achieve a decreased corrosion rate.

## 3. Materials and Methods

### 3.1. Electrodeposition of Zn–Ni–chitosan Coatings on Carbon Steel

Deposition of Zn–Ni–chitosan coatings were performed on carbon steel at room temperature in sulfate electrolytes with gradient concentrations of chitosan ((C_6_H_11_NO_4_)_n_, Cat. No. C8320, Solarbio, Beijing, China). The electrolytes were freshly prepared from distilled water using analytical grade reagents to prepare highly pure solutions. The sulfate electrolyte used was E_0_ = 250 g·L^−1^ ZnSO_4_•7H_2_O + 250 g·L^−1^ NiSO_4_•6H_2_O + 80 g·L^−1^ Na_2_SO_4_ + 26 g·L^−1^ H_3_BO_3_; E_CS1_ = E_0_ + 0.2 g·L^−^ chitosan; E_CS2_ = E_0_ + 0.6 g·L^−1^ chitosan; and E_CS3_ = E_0_ + 1.0 g·L^−1^ chitosan.

The Zn–Ni–chitosan coatings were electrodeposited on 20# carbon steel specimens with sizes of 50 mm × 13 mm × 2 mm. After grounding up to 2000#, the specimens were cleaned in ethyl alcohol using ultrasound for 15 min. The specimens were then used as substrates for the coatings and the cathodes after electrodeposition. A pure Ni specimen with a size of 50 mm × 20 mm × 5 mm was used as the anode. To record potential variations, a saturated calomel reference electrode (SCE) was also used as the reference electrode. The current density was controlled to be 90 mA cm^−2^ with a DJS-292E potentiostat during deposition. The electrodeposition time was controlled to be 733 s. When the electrodeposition finished, the coatings obtained from E_0_, E_CS1_, E_CS2,_ and E_CS3_ were named A_0_, A_CS1_, A_CS2_, and A_CS3_, respectively. The resultant coupons were washed with deionized water. Three duplicate samples were used for the following tests.

During the deposition, potential variations were monitored and recorded. The cathode current efficiency *η*_c_ was calculated based on the coupon mass measurement with an analytical balance. According to Equation (1), *η*_c_ was calculated [32]. One-way analysis of variance (ANOVA) was conducted using SPSS 13.0.

(1) $$\eta_{c}=\frac{2\left(w_{1}-w_{0}\right)F}{jSt[\omega_{\mathrm{Ni}}M_{\mathrm{Ni}}+(1-\omega_{\mathrm{Ni}})M_{\mathrm{Zn}}]},$$

where *η*_c_ is the cathode current efficiency, *w*_0_ is the mass of the carbon steel coupon in g, *w*_1_ is the mass of the coupon with the coating deposited in g, F is the Faraday constant, 96,485 C mol^−1^, *j* is the current density in mA cm^−2^, *S* is the depositing area in cm^2^, *t* is the deposition time in s, *ω*_Ni_ is the Ni content in at.%, *M*_Ni_ is the molar mass of Ni in g mol^−1^, and *M*_Zn_ is the molar mass of Zn in g mol^−1^.

### 3.2. Characterization of the Zn–Ni–chitosan Coatings

A surface characterization of the coatings was performed to investigate the influence of chitosan on the coatings. Energy dispersive spectroscopy (EDS) was performed on the coatings to test the Ni contents in the coatings. Scanning electron microscopy (SEM; S-3400N, Hitachi, Tokyo, Japan) with a beam voltage of 5 kV was conducted to observe the morphologies of the coatings. X-ray diffraction (XRD, D/max-Ultima IV diffractometer Rigaku, Tokyo, Japan) measurements were performed under the conditions of 40 kV, 30 mA, and graphite-filtered Cu Ka radiation (=0.1542 nm), to study the influence of chitosan on the crystalline structures of the coatings. Fourier transform infrared spectroscopy (FT-IR; Nicolet iN10 IR, Thermo Scientific, Waltham, MA, USA) was further conducted to detect the existence of chitosan in the coatings.

### 3.3. Investigation of the Interaction Between the Zn–Ni–chitosan Coatings and the SRB Medium

In this research, the sulfate-reducing bacteria strain *desulfovibrio caledoniensis*, separated from the rust layer of two-year-old Q235 carbon steel in the coastal areas of Qingdao, China [33], was used. The SRB was cultured in sterile Postgate’s C medium. The composition is shown in Table 1. Four-day-old SRB inoculum was inoculated into Postgate’s C medium with a volume fraction of 28%. Then the coatings were exposed for six days at 30 °C. During the incubation period, the pH was measured each day with a LEICI PHS-3C pH meter. The bacterial concentrations were also determined.

The corrosion behavior of the coatings was also investigated. The coatings surfaces were cleaned three times using 100 g·L^−1^ CH_3_COONH_4_ for 3 min at 70 °C to remove corrosion products and the biofilm (ISO 8407:1991). Then the corrosion rates were calculated according to Equation (2) and based on the weight loss measured by an analytical balance. The corrosion morphology was observed by SEM (S-3400N, Hitachi, Japan) after removing the corrosion products:

(2) $$v=\frac{m_{1}-m_{2}}{At\rho_{A}},$$

where *v* is the corrosion rate of the coating in mm y^−1^, *m*_1_ is the mass of the coupon with the coatings before exposure in g, *m*_2_ is the mass of the coupon with the coatings after exposure in g, *A* is the surface area exposed to SRB in mm^2^, *t* is the exposure time in y, and *ρ*_A_ is the Zn–Ni alloy density in g mm^–3^.

All containers and media were sterilized at 121 °C for 30 min. All operations were performed on an AIRTECH clean bench. Before the experiment, 30 min of ultraviolet light sterilization was conducted to provide a sterile environment. Three duplicate samples were used in these tests. One-way analysis of variance (ANOVA) and paired t-test were conducted using SPSS 13.0.

### 3.4. Antibacterial Tests on Zn–Ni–chitosan Coatings in E. coli Media

For the antibacterial experiment, the *E. coli* strain JM109 (sourced from Hong Kong University) was cultivated at 37 °C for 12 h in a Luria-Bertani (LB) medium (pH adjusted to 7), which is shown in Table 2. After culture, the bacterial concentration in the medium was measured with colony-forming units (CFUs) [34]. Then the bacterial body was separated from the medium by centrifugation of 4000 rpm for 5 min. The separated bacteria bodies were then suspended in 0.1 M phosphate buffer saline (PBS) (8.0 g·L^−1^ NaCl, 0.2 g·L^−1^ KCl, 1.44 g·L^−1^ Na_2_HPO_4_, and 0.44 g·L^−1^ KH_2_PO_4_ in distilled water). In the following experiments, the PBS medium suspended with 10^6^ cfu mL^−1^ *E. coli* was employed.

Specimens with coatings were exposed to a 10^6^ cfu mL^−1^ *E. coli* suspended PBS medium for 24 h. After being gently washed with sterile PBS, the specimens were then immersed in a 5% glutaraldehyde PBS medium for 30 min. Then the coatings were washed with sterile PBS and dyed in 1 μg mL^−1^ 4′,6-diamidino-2-phenylindole (DAPI) for 30 min to detect the bacterial coverage on the surface. Fluorescence microscopy was used for the fluorescent coverage calculation of the bacteria and it was performed at a magnification of 400×. The images captured by microscopy were analyzed by Image-pro Plus 6.0 software (Media Cybernetics, Rockville, MD, USA).

All media were prepared with analytical-grade reagents and distilled water, and then sterilized at 121 °C for 30 min before inoculation. All operations were performed on an AIRTECH clean bench (Suzhou, China). Before the experiment, 30 min of ultraviolet light sterilization was conducted to provide a sterile environment. Three duplicate samples were used in these tests. One-way analysis of variance (ANOVA) and paired t-test were conducted using SPSS 13.0.

## 4. Conclusions

In this research, Zn–Ni–chitosan coatings were obtained by the electrodeposition method. It was found that chitosan turned the electrodepositing potential strongly negative by adsorbing onto the depositing surfaces, which also led to the decrease of the cathodic current efficiency. The surface morphologies and Ni contents were obviously influenced by the chitosan addition. Further investigations showed that chitosan existed on the coating surfaces with effective structures. Due to the influence of the Ni content, A_CS1_ showed the highest corrosion resistance in the SRB medium. A_CS3_ revealed the best antibacterial property in the *E. coli*-suspended PBS medium, which may be attributed to the chitosan content in the coatings.

## Figures and Tables

**Table 1:** The composition of Postgate’s C medium.

Component	Content
KH_2_PO_4_	0.5 g
NH_4_Cl	1 g
CaCl_2_•6H_2_O	0.06 g
MgSO_4_•7H_2_O	0.06 g
70% Sodium lactate	6 mL
Yeast extract	1 g
Sodium citrate	0.3 g
Filtered seawater	1 L

**Table 2:** The composition of Luria-Bertani (LB) medium.

Component	Content
NaCl	10 g
Peptone from fish	10 g
Yeast extract	5 g
Distilled water	1 L
